# E3 ligase ligand optimization of Clinical PROTACs

**DOI:** 10.3389/fchem.2023.1098331

**Published:** 2023-01-17

**Authors:** Hanrui Jiang, Huan Xiong, Shuang-Xi Gu, Mingliang Wang

**Affiliations:** ^1^ Key Laboratory for Green Chemical Process of Ministry of Education, School of Chemical Engineering & Pharmacy, Wuhan Institute of Technology, Wuhan, China; ^2^ Zhongshan Institute for Drug Discovery, Shanghai Institute of Materia Medica, Chinese Academy of Sciences, Zhongshan, China; ^3^ Department of Medicinal Chemistry, Shanghai Institute of Materia Medica, Chinese Academy of Sciences, Shanghai, China

**Keywords:** PROTACs, E3 ubiquitin ligase ligand, structure optimization, clinical trials, CRBN

## Abstract

Proteolysis targeting chimeras (PROTACs) technology can realize the development of drugs for non-druggable targets that are difficult to achieve with traditional small molecules, and therefore has attracted extensive attention from both academia and industry. Up to now, there are more than 600 known E3 ubiquitin ligases with different structures and functions, but only a few have developed corresponding E3 ubiquitin ligase ligands, and the ligands used to design PROTAC molecules are limited to a few types such as VHL (Von-Hippel-Lindau), CRBN (Cereblon), MDM2 (Mouse Doubleminute 2 homolog), IAP (Inhibitor of apoptosis proteins), etc. Most of the PROTAC molecules that have entered clinical trials were developed based on CRBN ligands, and only **DT2216** was based on VHL ligand. Obviously, the structural optimization of E3 ubiquitin ligase ligands plays an instrumental role in PROTAC technology from bench to bedside. In this review, we review the structure optimization process of E3 ubiquitin ligase ligands currently entering clinical trials on PROTAC molecules, summarize some characteristics of these ligands in terms of druggability, and provide some preliminary insights into their structural optimization. We hope that this review will help medicinal chemists to develop more druggable molecules into clinical studies and to realize the greater therapeutic potential of PROTAC technology.

## 1 Introduction

The degradation of most damaged and soluble misfolded proteins is achieved by the 26S proteasome through ubiquitin-proteasome system (UPS)-mediated protein degradation (Paiva et al., 2019; Pohl et al., 2019; Kawahata et al., 2020). In the UPS, the proteasome conjugated to a protein substrate through enzymatic cascade (Hershko et al., 1983; Hershko et al., 1992; Komander et al., 2012). First, E1 (Ub activating enzyme) binds Ub (ubiquitin) *via* an ATP-dependent mechanism and then transfers Ub to E2 (Ubconjugating enzyme) by forming an E2 ubiquitin conjugate (Schulman et al., 2009; Ye et al., 2009). Next, the E3 (Ub ligase) mediates the transfer of the ubiquitin from E2 to the substrate protein, followed by 26S proteasome-induced degradation or post-translational modification of the substrate protein (Zheng et al., 2017). E3 ligase mediates the specificity of protein substrates through a non-covalent or covalent mechanism, and the type of E3 ligase determines the outcome of the substrate protein. For instance, TRAF6 (tumor necrosis factor receptor-associated factor 6) interacts with YAP (Yes-associated protein) and promotes its ubiquitination to enhance YAP stability (Liu et al., 2020). c-Cbl (Casitas B lymphoma) binds to the intracytoplasmic tail of PD-1 and targets it for ubiquitination-proteasomal degradation in macrophages, resulting in downregulation of PD-1 and reduced surface expression leading to increased tumor phagocytosis and tumor suppression (Lyle et al., 2019).

Proteolysis targeting chimeras (PROTACs) is based on proteasomes (Schapira et al., 2019). These bifunctional molecules consist of three parts: An E3-recruiting ligand, a POI (protein of interest) targeting warhead, and a linker connecting the two ligands (Sun et al., 2019). PROTAC degraders mediate their own formation of POI-PROTAC-E3 complexes with substrate proteins and E3 ubiquitin ligases, which lead to the degradation of the substrate protein *via* UPS (Wang et al., 2020; Yang et al., 2021). There are many target-based POI warheads and linkers available for the design and optimization of PROTAC degraders for medicinal chemists, but only a few E3 ubiquitin ligases ligands have been developed (Sun et al., 2019).

Arvinas is the first company to clinically implement two PROTAC degraders, the androgen receptor (AR) degrader **ARV-110** and the estrogen receptor (ER) degrader **ARV-471** (Mullard, 2019). The safety and effectiveness of **ARV-110** has been demonstrated in the treatment of metastatic castrated prostate cancer (mCRPC) (Neklesa et al., 2018; Neklesa et al., 2019; Gao et al., 2022), and **ARV-471** also has shown great potential in the treatment of breast cancer (Snyder et al., 2021). Since **ARV-110** and **ARV-471** entered clinic trials, an increasing number of protein targets have emerged to develop clinical degraders of PROTACs, such as BRD9 and IRAK4 (Table 1), while antitumor is currently the most concentrated field of research for PROTACs, except for KYMERA’s degrader **KT-474**, which is the only clinical degrader for autoimmune-disease (Békés et al., 2022). These PROTACs degraders are based on different E3 ligands, but mainly on CRBN-based ligands.

**TABLE 1:** PROTAC degraders in clinical development.

Company	Degrader	Target	E3 ligase	ROA	Highest phase	Clinical trial no. (if applicable)
Arvinas	ARV-110	AR	CRBN	Oral	Phase II	NCT03888612
Arvinas	ARV-766	AR	Undisclosed	Oral	Phase I	NCT05067140
Arvinas/Pfizer	ARV-471	ER	CRBN	Oral	Phase II	NCT04072952
Accutar Biotech	AC682	ER	CRBN	Oral	Phase I	NCT05080842
Bristol Myers Squibb	CC-94676	AR	CRBN	Oral	Phase I	NCT04428788
Dialectic Therapeutics	DT2216	BCL-X_L_	VHL	I.v	Phase I	NCT04428788
Foghorn Therapeutics	FHD-609	BRD9	Undisclosed	Oral	Phase I	NCT04965753
Kymera	KT-413	IRAK4	CRBN	I.v	Phase I	NA
Kymera	KT-333	STAT3	Undisclosed	Undisclosed	Phase I	NA
Kymera/Sanofi	KT-474	IRAK4	Undisclosed	Oral	Phase I	NCT04772885
Nurix Therapeutics	NX-2127	BTK	CRBN	Oral	Phase I	NCT04830137
Nurix Therapeutics	NX-5948	BTK	CRBN	Oral	Phase I	NCT04830137
C4 Therapeutics	CFT8634	BRD9	CRBN	Oral	IND- e	NA
C4 Therapeutics	CFT8919	EGFR^L858R^	CRBN	Oral	IND- e	NA
Cullgen	CG001419	TRK	CRBN	Oral	IND- e	NA

NA, not applicable.

**FIGURE 1:**
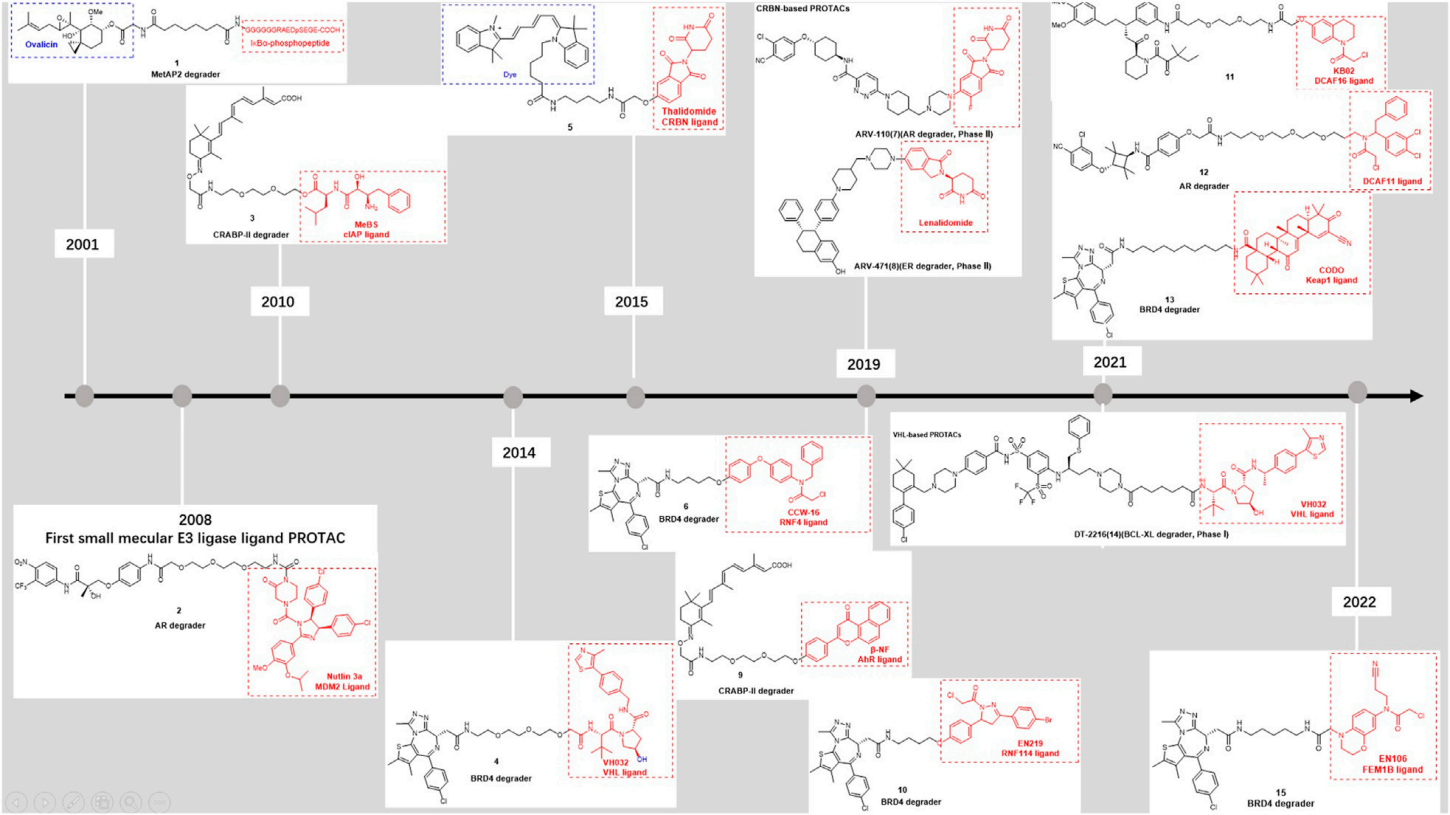
Timeline of E3 ligases ligands discoveries.

The first PROTAC was discovered in 2001 by Craig Crews, founder of Arvinas (Sakamoto et al., 2001) (Figure 1)*.* This compound consists of a covalent small molecule inhibitor of MetAP-2, and IκBα-phosphopeptide, enabling the ligase to ubiquitylate METAP2. As the first attempt to explore PROTACs, this compound exposed the poor cell permeability prevented it from being widely used, and the structure of phosphopeptides in this type of PROTAC is easily hydrolyzed by intracellular phosphatase, which reduces its stability. Therefore, the desired small molecule E3 ligase ligand, must have good membrane permeability, be stable *in vitro* environment and have strong affinity to E3 ubiquitin ligase, on the basis of which PROTACs will have stronger druggability.

In the past 2 decades, various E3 ligase ligands based on different functions have been reported. Such as MDM2 ligand (Honda et al., 1997; Schneekloth et al., 2008; Saadatzadeh et al., 2017), cIAP ligands (Sato et al., 2008; Itoh et al., 2011; Ohoka et al., 2017), VHL ligand (Schneekloth et al., 2004; Rodriguez-Gonzalez et al., 2008), CRBN ligand (Winter et al., 2015), AhR (Aryl hydrocarbon receptor) ligand (Ohtake et al., 2007; Ohoka et al., 2019), DCAF (DDB1- And CUL4-Associated Factor) 11 and 15 and 16 ligands (Han et al., 2017; Zhang et al., 2019; Zhang et al., 2021), RNF (RING finger protein) 4 and 114 ligands (Kumar et al., 2019; Ward et al., 2019; Luo et al., 2021), FEM1B (Fem-1 Homolog B) ligand (Henning et al., 2022), KEAP1 (Kelch Like ECH Associated Protein 1) ligand (Tong et al., 2020; Wei et al., 2021; Pei et al., 2022) (Figure 1). They were developed based on the function of different E3 ligases thus have different properties. For instance, VHL is the substrate receptor of CRL2VHL E3 ubiquitin ligase.

The Pro564 residue of HIF-1α (Hypoxia-Inducible Factor-1α) is hydroxylated by prolyl hydroxylase (also the hydroxyl group in the VHL ligands), bound to VHL proteins and subsequently ubiquitinated by CRL2VHL E3 (Ivan et al., 2001; Schneekloth et al., 2004). HIF-1α protects cells during hypoxia, and VHL-based PROTAC shows good degradation activity in most cases, and VHL ligands even reduce side effects in some cases (Jaakkola et al., 2001). However, in subsequent studies, the poor membrane permeability and low oral delivery rate of VHL ligands limited their application. The E3 ubiquitin ligase ligands are like a toolbox for PROTACs, and the appropriate “tool” is selected based on the different properties of E3 ligands for the purpose of the researchers.

In this review, we will summarize the experience of small molecule PROTACs currently entering clinical trials in optimizing E3 ligase ligands for degraders from the perspective of chemical structure. It is expected to shed light on the optimization of E3 ligase ligands for degraders of PROTACs in the future.

## 2 E3 ligase ligands optimization of PROTACs for clinical application

### 2.1 E3 ligase ligands optimization of AR PROTACs

Annually, more than 350,000 deaths are associated with prostate cancer, making the disease one of the leading causes of cancer-related death in men, and the androgen receptor (AR) is believed to drive hormone dependency of prostate cancer (Rebello et al., 2021). Key AR gene alterations contribute to castration-resistant prostate cancer (CRPC) (de Bono et al., 2020). Both enzalutamide and abiraterone have shown good results as AR antagonists in the treatment of prostate cancer. However, the drug resistance is inevitable when mutations occur in the ligand-binding domain (Teo et al., 2019). Therefore, the development of AR degraders based on PROTACs technology has become a new strategy (Pan et al., 2007).

**FIGURE 2:**
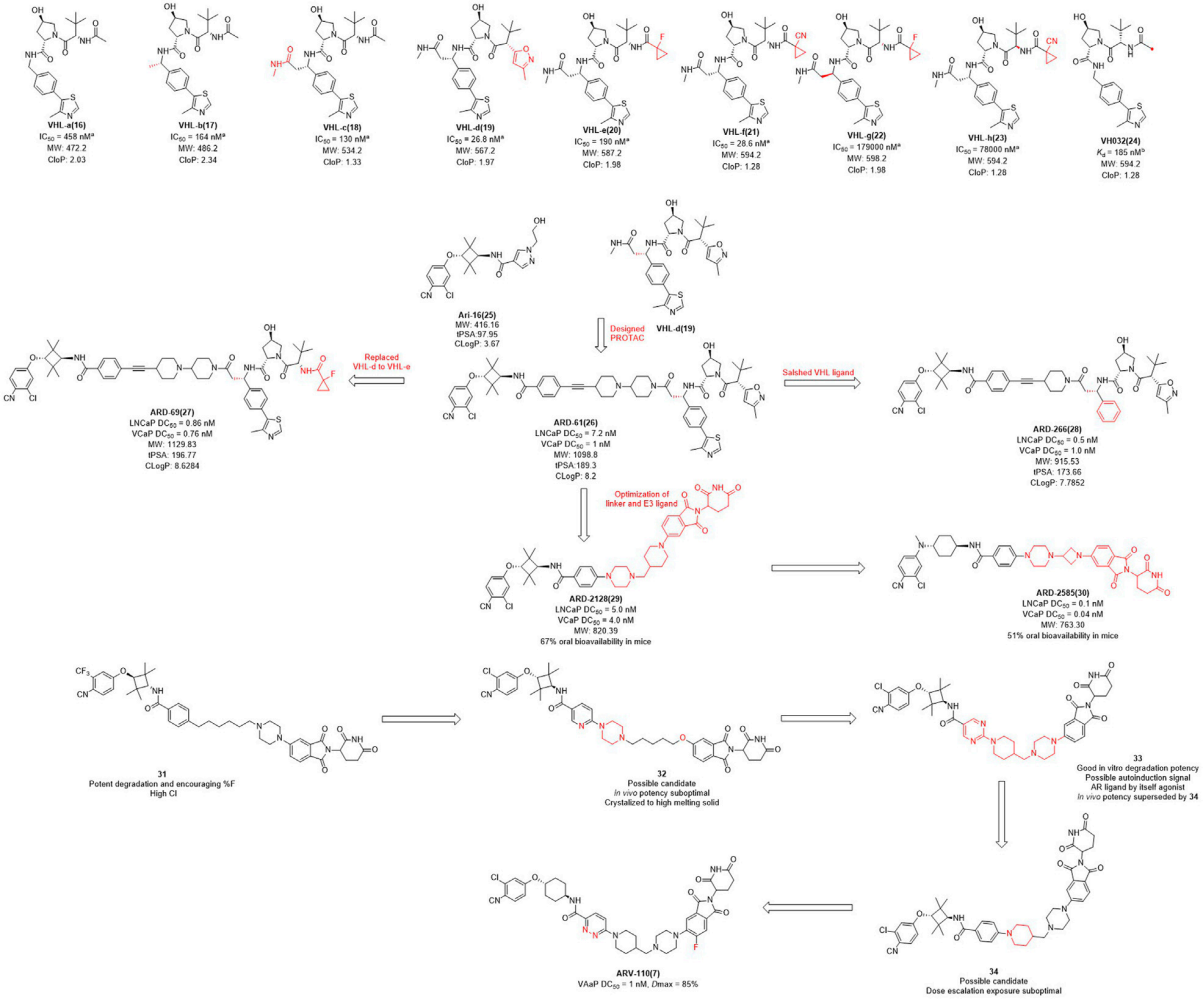
Chemical structures of VHL ligands and their binding affinities to VHL protein and the design and optimization of AR PROTACs. ^a^Inhibitory activity (IC50) of E3 ligands on their substrates. ^b^inding affinity (Kd) of E3 ligands to their respective substrates MW, molecular weight; tPSA, total polar surface area; cLog P, calculated Log P; *The arrows in the figure do not mean progressive relationship between these compounds.

The earlier AR PROTAC was designed using enzalutamide as the AR antagonist and VHL-b ligands as E3 ligase ligand. In 2019, Wang et al. selected **Ari-16** as the antagonist of the degrader by screening various VHL-based E3 ligase ligands **(compounds 16–24)** to build different AR degraders (Kregel et al., 2020; Zhao et al., 2020) (Figure 2). During E3 ligase ligand optimization process, they found that the (*S*)-methyl group in **VHL-b** exposed to the solvent environment could serve as a possible tethering point for the design of AR degraders (Han et al., 2019). Subsequently, they reported that **ARD-61** is an AR degrader with DC_50_ (concentration that resulted in a 50% targeted protein degradation) values of 7.2 nM and 1.0 nM in LNCaP and VCaP cells, respectively. Apparently, the large molecular weight of the VHL ligand makes ARD-61 too large (MW = 1095.8) for its degradation activity to be significant. In their following work, the E3 ligase ligand part of **ARD-61** was replaced with a VHL ligand and the optimized degrader **ARD-69** showed AR DC_50_ values of 0.86 nM and 0.76 nM in LNCaP and VCaP cells, respectively. However, the molecular weight of **ARD-69** was still too large as an AR degrader. Subsequently, they shortened the linker length and modified the VHL ligand to decrease its molecular weight. Thus, led to degrader **ARD-266** with similar degradation activity, the AR DC_50_ values were 0.5 nM and 1.0 nM in LNCaP and VCaP cells, respectively (Han et al., 2019). The poor membrane permeability and low oral availability of VHL ligands led to its eventual replacement by CRBN ligands which is also used in Bristol Myers Squibb’s ER clinical degrader **CC-94676**. On the basis of the above three molecules, they discovered that **ARD-2128** showed the same degradation activity as **ARD-61** (MW = 820.4), but its molecular weight was significantly reduced and its bioavailability in mice reached 67% (Han et al., 2021). Finally, on the basis of CRBN ligand, they re-optimized the linker and the antagonist and disclosed **ARD-2585**, which has 51% oral bioavailability in mice and with AR DC_50_ of 0.10 nM and 0.04 nM in LNCaP and VCaP cells, respectively (Xiang et al., 2021).

In the pharmaceutical industry, AR degraders have also received the attention of Arvinas, whose CRBN-based AR degrader **ARV-110** (Figure 2) is currently in phase II clinical trials (Snyder et al., 2021). Arvinas has also performed many optimizations for AR degradation. First of all, compounds **31** and **32** showed good degradation abilities *in vitro*, but also with a high clearance rate *in vivo*. Subsequently, they disclosed that bi-functional compound **33** showed strong degradation activity *in vitro*, however, poor activity *in vivo*, perhaps due to the metabolism of **33**. The structure of compound **34** was optimized on the basis of compound **33** with improved activity *in vivo*, but it was dose dependent and needed further optimization. Finally, the H at position 3 of the benzene ring of the CRBN ligand of **34** was replaced with fluorine to obtain degrader **ARV-110** which improved the druggability. **ARV-110** was highly efficient in VCaP cells with a DC_50_ value with 1 nM and *D*max (maximal levels of protein degradation) of 85% (Snyder et al., 2021). Above all, the E3 ubiquitin ligand functions as the promoter of the degradation process, and optimizing the E3 ligand and modifying its molecular structure is expected to improve the activity and oral delivery rate of the degraders.

### 2.2 E3 ligase ligands optimization of ERα PROTACs

**FIGURE 3:**
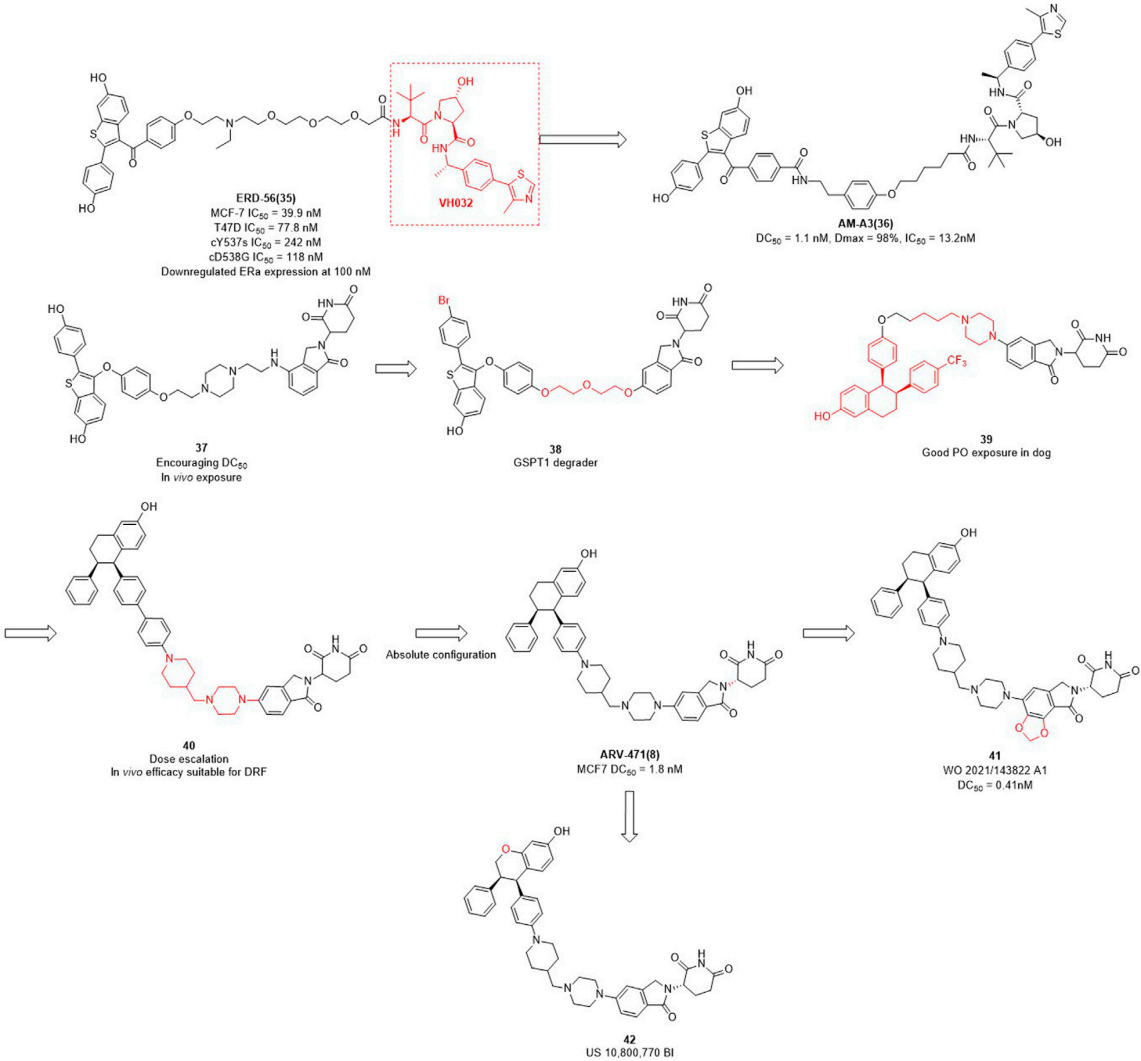
Design and optimization process of ER PROTACs.

The importance of estrogen regulates (ER) for female breast cancer is similar to AR for male prostate cancer. ER targets are associated with 70%–80% of breast cancer profiles, and become the primary therapeutic target for this disease (Ohoka et al., 2018; Waks et al., 2019; Criscitiello et al., 2022). ERα is a member of the nuclear receptor family and plays a crucial role in mediating the estrogen signaling pathway within the mammary glands and female reproductive tract (Arnal et al., 2017). In contrast to the high expression of ERα in breast tumors, the ERα expression is low in normal breast epithelium cells (Huang et al., 2014). ERα knockout experiments in rats demonstrated that ERα plays an important role in promoting the formation of breast cancer cells in the mammary gland (Zhang et al., 2011). In addition to ERα knockout, PROTAC can also reduce the expression levels of ERα in breast. Wang et al. focused not only on AR degraders but also on ERα degraders. In their initial studies of ERα degradation, they singled out raloxifene as ERα-binding ligand and CRBN and VHL as E3 ligase ligands, respectively. Interestingly, the VHL-based PROTACs were shown to induce significant degradation of the target protein, whereas no obvious protein degradation was observed for CRBN-based molecules. Therefore, they synthesized a series of PROTACs based on raloxifene and **VH032** (Figure 3). Among them, **ERD-56** showed significant degradation properties of ERα protein at 100 nM, and also showed good anti-proliferative activity in MCF-7 and T47D cells with IC_50_ (half maximal inhibitory concentration) of 39.9 nM and 77.8 nM (Gonzalez et al., 2020). In order to improve the potency of **ERD-56**, they optimized the linker and **ERD-308** showed the best efficacy with DC_50_ of 0.17 nM (Hu et al., 2019). Interestingly, the ER protein degraders reported above were all designed based on **VH032** as an E3 ligase ligand. Due to the poor druggability of **VH032** itself, the druggability of ER protein degraders developed based on it was also generally poor. Different from the above studies, Arvinas has also been working on the development of ERα degraders based on CRBN. First, they designed ERα degraders based on raloxifene and lenalidomide, and found that degrader **37** (Figure 3) showed good degradation activity. Based on **37**, they optimized the linker and ERα ligands to obtain the degrader **38** targeting GSPT1. Then they modified the structure of raloxifene to obtain degrader **39**, due to the poor druggability of the long-chain linker, they decided to use the same linker that existed in **ARV-110** to obtain degrader **40**. From the activity screening experiment, it was found that the chiral degrader **ARV-471** had better degradation activity than **40**. The ERα degradation activity DC_50_ in MCF-7 cells was 1.8 nM and thus **ARV-471** became another clinical degrader of Arvinas (Snyder et al., 2021). Hengrui Medicine further optimized CRBN E3 ligase ligand and improve the degradation activity of ER degraders, and found compound **41** with DC_50_ value of 0.41 nM in MCF-7 cells (Yang et al., 2021). Accutar Biotech undisclosed their structure of ER clinical degrader (AC682) (NCT05080842), in their research they found that the compound **42** with DC_50_ value of 0.3 nM in MCF-7 cells (Fan et al., 2021). In summary, the optimization of E3 ubiquitin ligase ligand is the key to improve the oral availability and potency of PROTACs.

### 2.3 E3 ligase ligands optimization of BTK PROTACs

**FIGURE 4:**
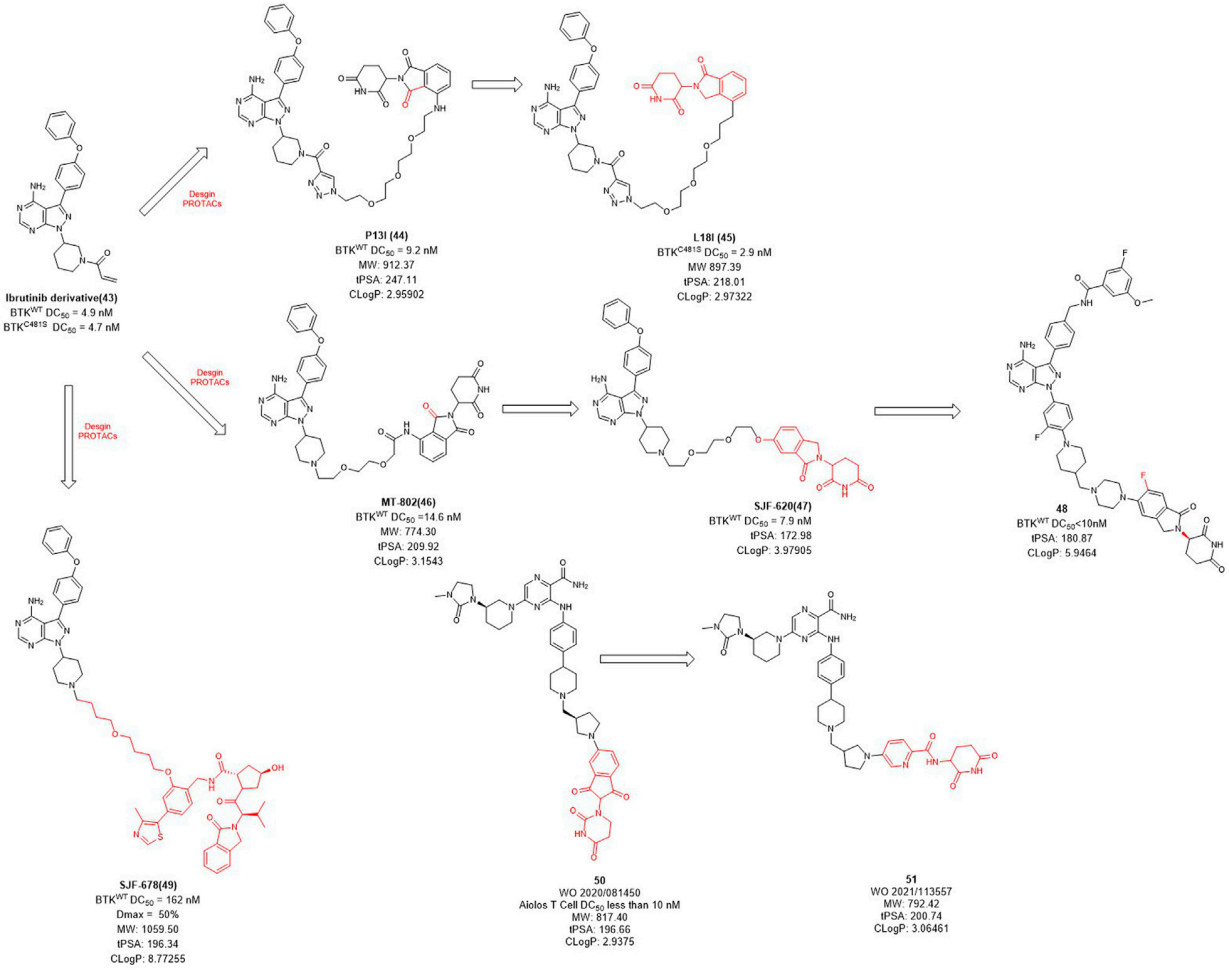
Design and optimization of BTK PROTACs.

Bruton’s tyrosine kinase (BTK) is highly expressed in various of lymphoma cells and plays an essential role in B-cell receptor (BCR) signal and B cell activation (Davis et al., 2010). Since the ATP binding site of BTK is highly conserved, how to achieve kinase selectivity of BTK inhibitors becomes the key issue. Ibrutinib is the first approved BTK covalent inhibitor with high selectivity, strong activity and good oral bioavailability (Pan et al., 2007). The acrylamide warhead of ibrutinib forms a covalent bond with the sulfhydryl group of the cysteine residue 481 in BTK, which is irreversible and thus permanently inactivates BTK kinase with an IC_50_ value of 0.5 nM after 2 h (Pan et al., 2007). However, the C481S BTK mutation (cysteine to serine mutation at position 481) prevents the formation of the critical covalent bond with ibrutinib, leading to drug resistance (Woyach et al., 2014). In order to overcome this challenge, in 2018, Rao et al. applied the PROTAC technology for ibrutinib-resistant BTK degradation and reported **P13I** (Figure 4) which is an ibrutinib and pomalidomide-linked degrader with DC_50_ value of 9.2 nM for wild-type and DC_50_ value of 30 nM for ibrutinib-resistant C481S BTK in Mino cells (Sun et al., 2018). While ibrutinib has difficulty inhibiting the autophosphorylation of C481S mutant BTK, **P13I** is effective at low concentrations. For HBL-1 cells expressing the C481S mutant BTK, the GI_50_ (50% growth inhibitory concentration) of **P13I** was about 28 nM compared to about 700 nM for ibrutinib, a 20-fold decrease in potency. In addition, **P13I** showed no effect on ITK, EGFR and TEC family kinases that cause side effects. Subsequently, in order to improve the aqueous solubility of **P13I** for both *in vitro* and *in vivo* evaluations, Rao et al. further optimized the E3 ligase ligand of **P13I** with lenalidomide and to obtain a new degrader **L18I** (Sievers et al., 2018; Sun et al., 2019). **L18I** exhibited good solubility in phosphate buffered saline (PBS), and inhibit C481S BTK in DLBCL tumors growth *in vivo*. These efforts suggest that PROTACs may provide a new treatment strategy for ibrutinib-resistant tumors.

Unlike the usually used ibrutinib-based BTK PROTACs (In this section we only discuss the reversible non-covalent BTK PROTACs), Crews et al. reported another non-covalent analog of ibrutinib, and developed a novel CRBN-recruiting BTK PROTAC, **MT-802** (Figure 4), which induced the efficient degradation of both wild-type (DC_50_ = 14.6 nM) and C481S mutation (DC_50_ = 14.9 nM) BTK (Buhimschi et al., 2018). Meanwhile, they found that the degradation efficiency of VHL-recruited BTK PROTAC degrader **SJF678** was significantly weaker than that of CRBN-recruited BTK PROTACs. They then developed a BTK degrader **49** based on the E3 ligand of **ARV-110** with DC_50_ value less than 10 nM treated in RAMOS cell lines for 6 h. Nurix Therapeutics discovered two BTK degraders, NX-2127 and NX-5948, currently in Phase I clinical trials and their structures have not been disclosed. In recent years, they have disclosed oral PROTAC degraders **50** and **51** with good activities both *in vitro* and *in vivo*, as well as oral bioavailability (Robbins et al., 2020; Sands et al., 2020). The druggability of BTK degraders was significantly improved after modifying the linker to make it more rigid and reducing the molecular weight of the E3 ligand.

### 2.4 E3 ligase ligands optimization of IRAK4 PROTACs

**FIGURE 5:**
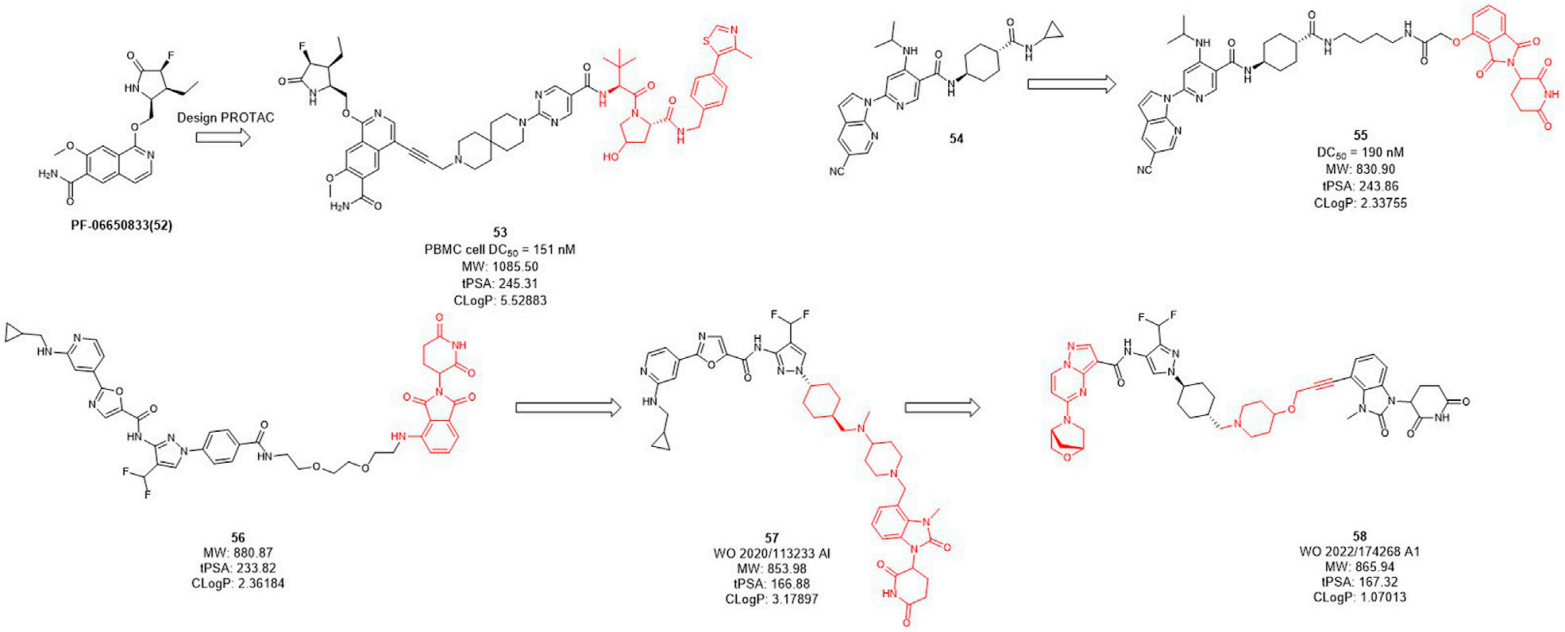
Design and optimization of IRAK4 PROTACs.

Interleukin-1 receptor-associated kinase 4 (IRAK4) is a serine/threonine kinase that not only performs phosphorylation but also functions as a scaffold role in Toll-like receptor (TLR) and interleukin-1 receptor (IL-1R) signaling pathways (Brzezinska et al., 2009; Lim et al., 2013; Vollmer et al., 2017). As a promising therapeutic target for diffusing large B-cell lymphoma driven by the MYD88 L265P mutant, the IRAK4 target receives significant attention. While Previous inhibitors had only moderate effects on IRAK4 target because they inhibited kinase function but had no effect on scaffold function. Unlike traditional small molecule inhibitors, which only inhibit kinase activity, PROTACs for protein degradation may offer a solution to block both IRAK4 kinase activity and scaffold capabilities. In 2019, Anderson et al. selected **PF-06650833** as IRAK4 inhibitor and synthesized a series of compounds based on VHL, CRBN, and IAP ligands. Among them, only the degrader **53** (Figure 5) based on VHL showed degradation activity of IRAK, with the DC_50_ value of 151 nM in PBMC cell (Nunes et al., 2019). In 2020, Dai et al. designed and synthesized a series of CRBN-based IRAK4 degraders and compound **55** showed DC_50_ value of 190 nM *in vitro* (Zhang et al., 2020). However, its degradation activity may be weaker than that of inhibitors **54**. It may be due to the weak affinity between the inhibitor and the target protein, and replacing the E3 ligand will not obtain the desired potency.

Kymera has two IRAK4 degraders, KT-474 and KT-413 in phase I clinical trial while their structures are undisclosed (Kymera, 2022). From their published patent (WO 2020/113233 Al), they obtained a series of IRAK4 degraders based on CRBN in combination with different IRAK4 inhibitors, most of which such as compound **56** (Figure 5) showed more than 50% degradation of IRAK4 at 0.01 nM in PMBC cells (Kargbo, 2019) (e.g., compound **57**). Subsequently, the E3 ligase ligand part of **56** was replaced with the CRBN ligand, and the linker-optimized degrader **57** also showed DC_50_ less than 0.01 nM in PMBC cells (Mainolfi et al., 2020). Recently, they disclosed the structure of compound **58** which is also based on CRBN ligand and showed excellent *in vitro* and *in vivo* activity, as well as oral bioavailability (Gollob et al., 2022).

### 2.5 E3 ligase ligands optimization of TRK PROTACs

The tropomyosin receptor kinase (TRK) receptor family comprises three members: TRKA, TRKB, and TRKC that are encoded by the NTRK1, NTRK2, and NTRK3 genes, respectively, which plays an important role in regulating cell differentiation, proliferation, pain, and survival (Pulciani et al., 1982; Martin-Zanca et al., 1986; Klein et al., 1989). TRKs are tyrosine kinases receptors and their main implication is the development and function of neuronal tissues (Kargbo, 2020). Although the targeted treatment of TRK1 and TRK2 shows an overall good safety profile in the clinic trials, this strategy could also be improved because currently available pan-TRK kinase inhibitors may induce off-target adverse effects by modulating TRK family members present in the CNS. Currently, moderate off-target adverse effects have been observed, such as dizziness/ataxia, paresthesia, and weight gain (Drilon, 2019). Non-specific side effects and drug resistance to TRK kinase inhibitors remain great challenges for effective treatment (Kargbo, 2020). In contrast, PROTACs technology keep the target protein in the periphery without penetrating the blood-brain barrier, thus avoid the side effects of off-targeting to the CNS.

**FIGURE 6:**
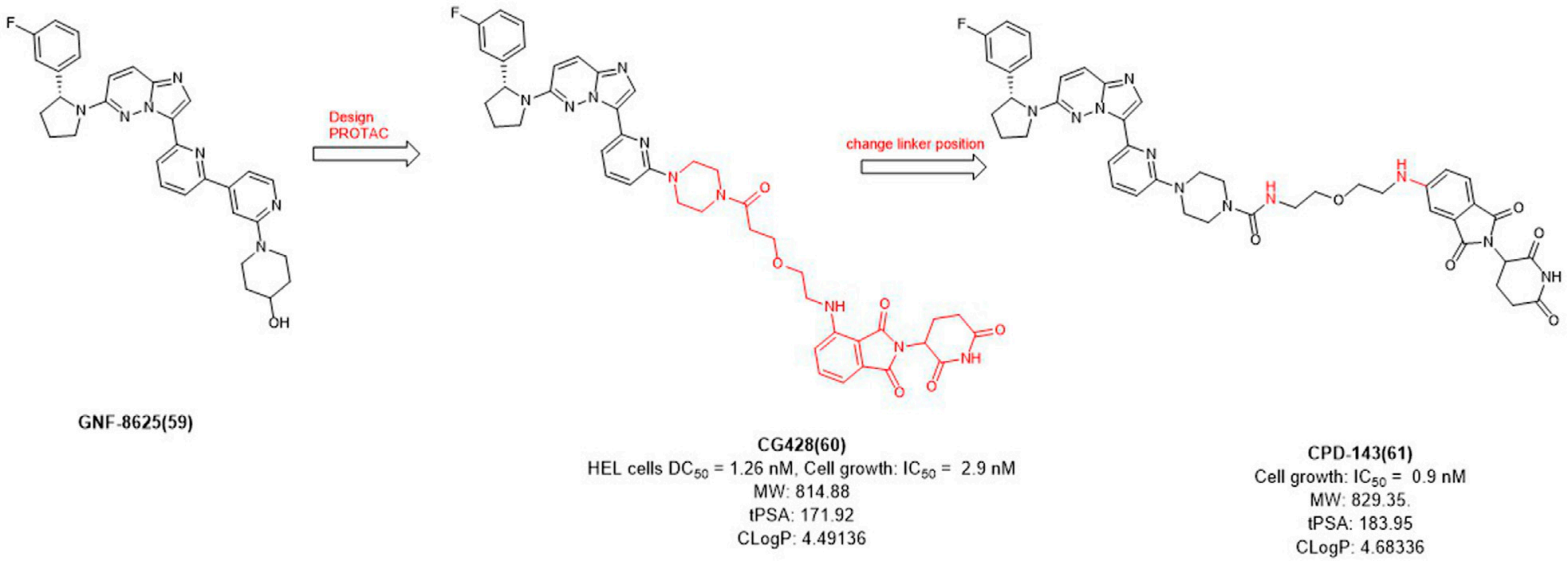
Design and optimization of TRK PROTACs.

In 2020, Cullgen et al. selected **GNF-8625** (Figure 6) as TRK inhibitor and linked with CRBN to obtain a series of TRK degraders and **CG428** proved to be the most promising degrader. It demonstrated that **CG428** can induce the degradation of wild-type TRKA in HEL cells with DC_50_ value of 1.26 nM and also inhibit cell growth with IC_50_ value of 2.9 nM (Chen et al., 2020). They then changed the connect position of pomalidomide with linker and obtained compound **CPD-143** with an increased activity which the cell growth IC_50_ was 0.9 nM (Kargbo, 2020). It can be seen that when optimizing PROTACs, once the POI and E3 ligands were identified, changing the linker position on the E3 ligase ligand could be considered to improve the potency of PROTACs.

### 2.6 E3 ligase ligands optimization of BRD9 PROTACs

The BRD9 (bromodomain-containing protein 9) has gained special attention as a component of the human ATP-dependent chromatin remodeling BAF (BRG1/BRM-associated factor) complex (also known as mammalian SWI/SNF (SWItch Sucrose Non-Fermentable)) (Kadoch et al., 2013; Theodoulou et al., 2016). Studies have shown that BRD9 is preferentially used by cancers harboring SMARCB1 abnormalities, such as malignant rhabdoid tumors and several specific types of sarcomas (Kim et al., 2014). BRD9-containing complexes bind to active promoters and enhancers where they contribute to gene expression (Gatchalian et al., 2018). Loss of BRD9 leads to changes in gene expression related to apoptosis regulation, translation and development regulation. BRD9 is essential for the proliferation of SMARCB1-deficient cancer cell lines and is therefore a therapeutic target for these lethal cancers, and it is also a key target for causing acute myeloid leukemia (Hohmann et al., 2016; Michel et al., 2018). Despite the early discovery of BRD9 inhibitors, there is limited understanding of the function of BRD9 beyond acetyl lysine recognition based on early chemical probes.

**FIGURE 7:**
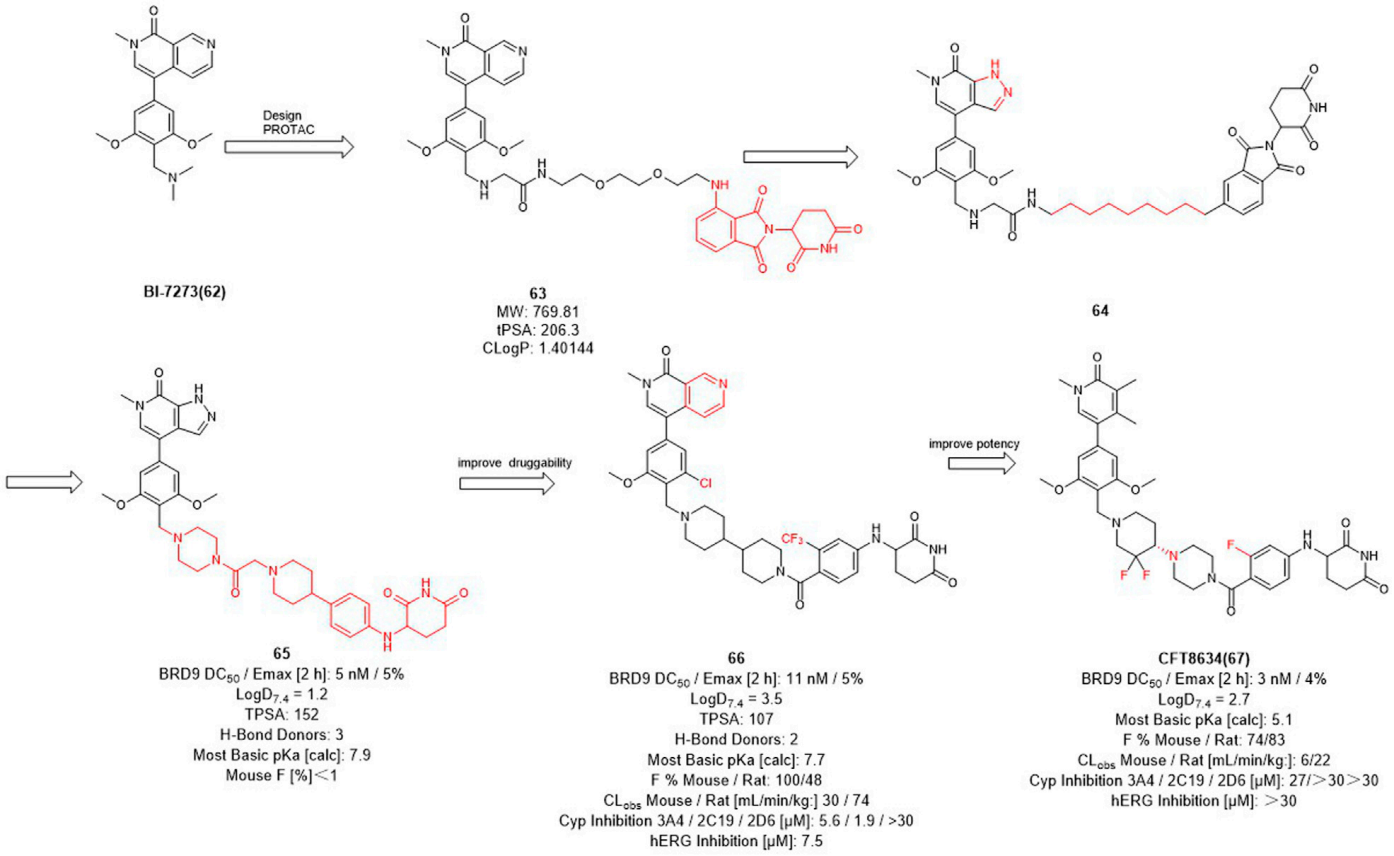
Design and optimization of BRD9 PROTACs.

Therefore, Bradner et al. designed the first BRD9 degrader **63** in 2017 to provide a tool compound (Figure 7) (Remillard et al., 2017). Compound **63** was designed by using **BI-7273** as inhibitor of BRD9 and CRBN ligand pomalidomide as E3 ligase ligand, it turned out to be valuable for exploring the biological and therapeutic potential of degrading BRD9. C4 Therapeutics (C4T) started with **64** and optimized both BRD9 inhibitors and E3 ligands and finally obtained the tool degrader **65** with DC_50_ value of 5 nM.

Compound **66** was optimized based on the structure of compound **65** with fewer hydrogen bond donors and its bioavailability (F %) in mice was increased to 100%, thus improving the druggability of the degrader. Subsequently, they made minor modifications of the POI and linker of compound **65**, more importantly, for the E3 ligase ligand part, they replaced the F to trifluoromethyl group of the pomalidomide to obtain the degrader **CFT8634** with high oral bioavailability (Jackson et al., 2022). Finally, Food and drug Administration (FDA) has granted orphan drug designation (ODD) to **CFT8634** (Figure 7) for the treatment of soft tissue sarcoma which is an orally bioavailable, selective degrader of BRD9 (DC_50_ = 3 nM) (Sabnis, 2021). In summary, the optimization process from **64** to **CFT8634** indicates that the dramatic change in their E3 ligands fraction improves their treatment potential.

### 2.7 E3 ligase ligands optimization of EGFR L858R PROTACs

**FIGURE 8:**
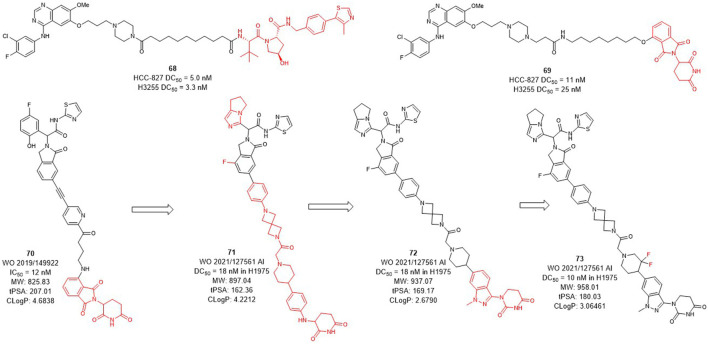
Design and optimization of EGFR L858R PROTACs.

Several epidermal growth factor receptor (EGFR) tyrosine kinase inhibitors have been developed and approved by the FDA for the treatment of non-small-cell lung cancer, but their efficacy may be compromised by drug resistance in EGFR-mutant variants (Sharma et al., 2007; Hirsch et al., 2017). Activating mutations, mainly in-frame deletions in exon 19 and L858R mutations, the former occurring in the αC-helix domain and the latter in the adenosine triphosphate (ATP) binding domain of the EGFR kinase. The EGFR L858R variant leads to poor prognosis and high incidence of malignant pleural effusion in non-small cell lung cancer, and the current small molecule drugs are only moderately effective against this variant (Kohno et al., 2021; Matsui et al., 2021). The development of EGFR L858R degraders based on PROTACs technology has become a new strategy. In 2020, Jin et al. designed and synthesized EGFR degraders with gefitinib and VHL or CRBN-recruited E3 ligands. The DC_50_ values of VHL-based degrader **68** (Figure 8) were 5.0 nM in HCC-827 cells and 3.3 nM in H3255 cells. The DC_50_ values of compound **69** which was based on CRBN ligand were 11 nM and 25 nM, respectively. In addition, they also showed good plasma exposure in mice.

C4T discovered the CRBN-based EGFR L858R degrader **70** (Figure 8) with an IC_50_ (BaF3 EGFR T790M/L858R/C797S degradation) value of 12 nM (Duplessis et al., 2019). After the optimization of the linker and the CRBN ligand, compounds **71** and **72** were subsequently synthesized, both showed a DC_50_ value of 18 nM in H1975 cells. Based on the structure of **72**, compound **73** was further identified, which improved the DC_50_ value to 10 nM in H1975 cells. C4T replaced the lenalidomide ligand with a new CRBN-based derivative ligand, resulted in a better activity of compound **73** than compound **70** on the basis of structural optimization (Nasveschuk et al., 2021).

### 2.8 E3 ligase ligands optimization of BCL-X_L_ PROTACs

**FIGURE 9:**
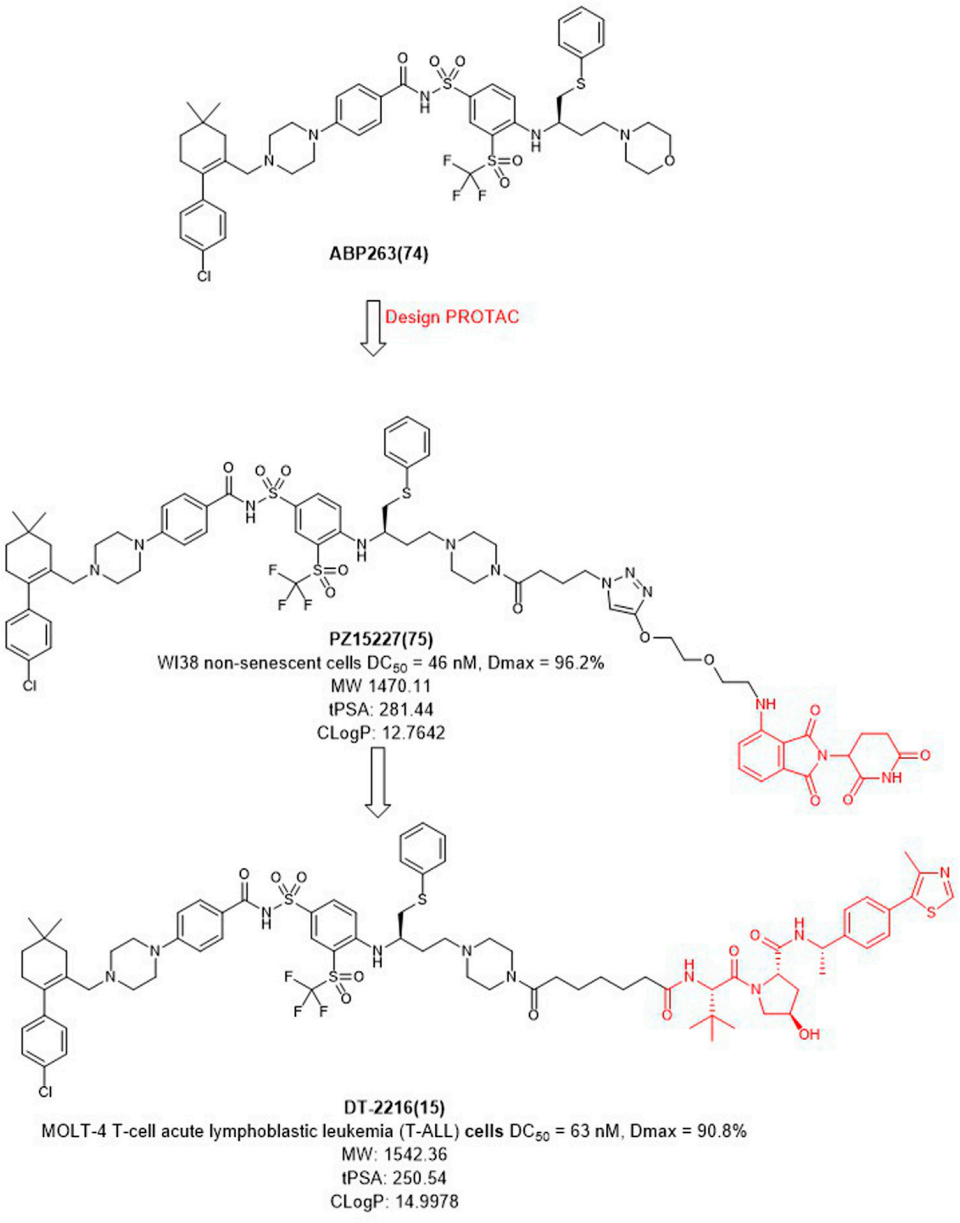
Design and optimization of BCL-X_L_ PROTACs.

BCL-X_L_ belongs to the anti-apoptotic BCL-2 protein family and plays a key role in determining cell life and death by regulating the intrinsic apoptotic pathway (Czabotar et al., 2014). The anti-apoptotic function of BCL-X_L_ protects cancer cells and induces drug resistance, which also promotes tumor progression (Igney et al., 2002). Inhibition of BCL-X_L_ has been of great interest as a potential cancer therapeutic strategy. However, traditional BCL-X_L_ inhibitors, such as **ABT263** (Figure 9), exhibit targeted and dose-limited platelet toxicity (Zhang et al., 2019). Since the tissue distribution studies of VHL and CRBN have shown that its expression in platelets is minimal, degraders of BCL-X_L_ could be developed through PROTAC technology.

In 2019, Zheng et al. selected **ABT263** (Figure 9) as BCL-X_L_ inhibitor connected with CRBN to obtain the degrader **PZ15227** with DC_50_ value of 46 nM and *D*max of 96.2% in WI38 non-senescent cells (NCs) (He et al., 2020). Compared with **ABT263**, **PZ15227** showed reduced toxicity to platelets but remained toxicity against senescent cells because CRBN was less expressed in platelets. Subsequently, they found that CRBN-based PROTACs were highly potent against other cancer cell lines, but less potent in MOLT-4 cells, possibly due to the low expression of CRBN (Zhang et al., 2020).

In 2021, Zheng *et al.* discovered **DT2216** (NCT04886622) as an effective BCL-X_L_ degrader based on VHL E3 ligase with a DC_50_ value of 63 nM and *D*max of 90.8% in MOLT-4 cells. Compared with **ABT263** (EC_50_ = 0.191 μM, half max effective concentration) and **DT2216** (EC_50_ = 0.052 μM), the latter showed increased cytotoxicity to MOLT-4 cells. More importantly, **DT2216** exerted almost no effect on the viability of platelets up to a concentration of 3 μM which showed better effect than **PZ15227**. **DT2216** was found to have enhanced efficacy against a variety of BCL-X_L_-dependent leukemia cell lines and exhibited much less toxic to platelets than **ABT263** (Khan et al., 2019). Therefore, **DT2216** was approved by FDA to enter phase I clinical trials for the treatment of advanced liquid and solid tumors. These findings demonstrated the potential of using PROTAC to reduce the toxicity of targeted drugs.

### 2.9 E3 ligase ligands optimization of STAT3 PROTACs

**FIGURE 10:**
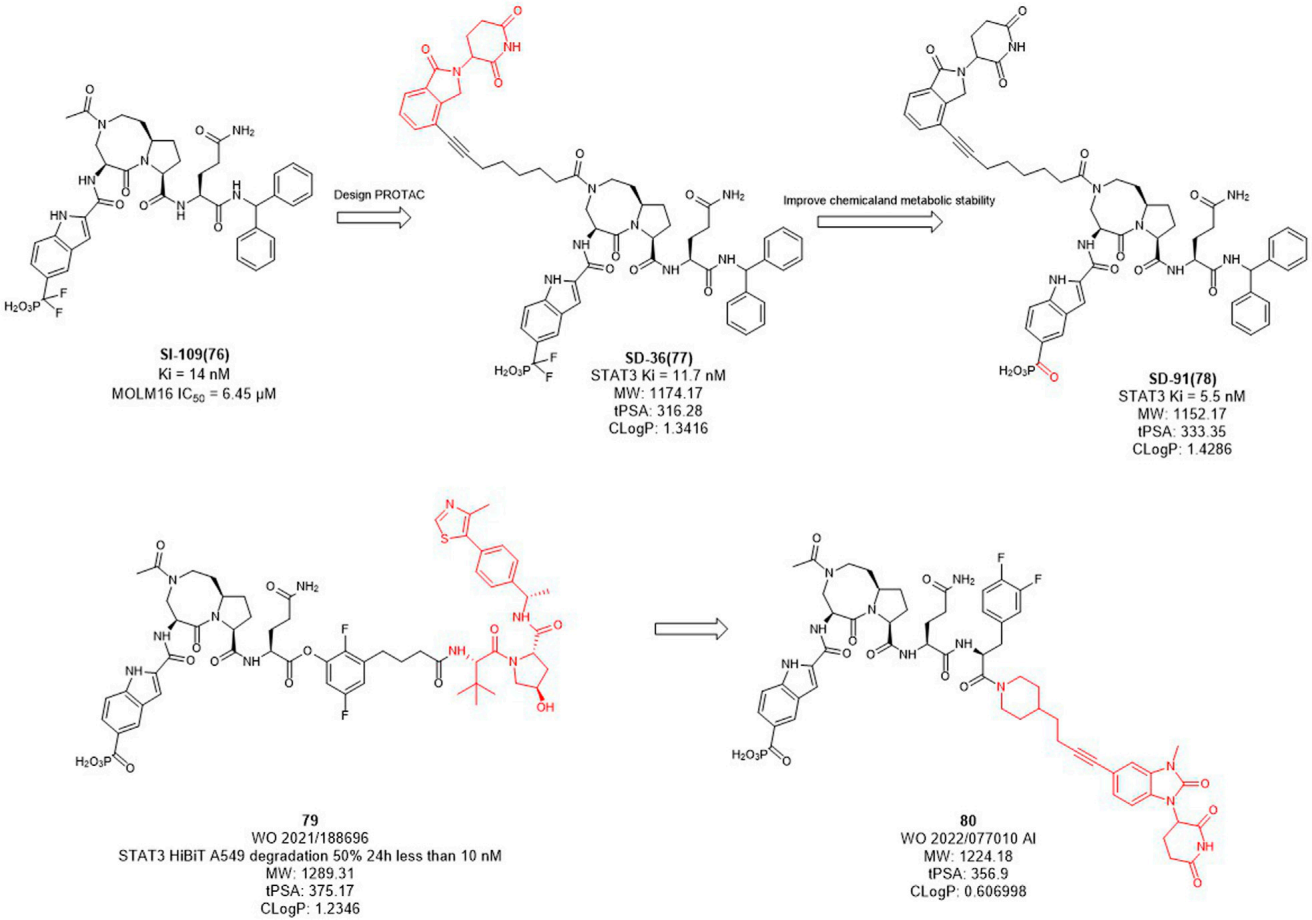
Design and optimization of STAT3 PROTACs.

In mammalian cells, the signal transducer and activator of transcription 3 (STAT3) is an essential component of the seven members of the STAT family (STAT1, 2, 3, 4, 5a, 5b, 6). STAT3 is widely expressed in a variety of cells and tissues and activates the expression of downstream genes in response to various cytokines, growth factors and other signals (Yu et al., 2009). Under normal physiological conditions, STAT3 activation is rapid and transient, mainly due to the presence of negative regulators in cells. However, STAT3 is continuously activated and expressed at high levels in tumor cells. Overexpression of STAT3 is strongly associated with cancer cell survival, proliferation, invasion, metastasis, drug resistance, and immune evasion, among other related genes. Since STAT3 dysregulation contributes to many human cancers and other human diseases (Wang et al., 2018). Inhibition or downregulation of STAT3 expression has become a main strategy for cancer therapy. However, to date, no drugs based on STAT3 targets have been approved in the market. Wang et al. designed several STAT3 degraders based on **SI-109** (Figure 10) and lenalidomide, and discovered that degrader **SD-36** exhibited good degradation activity (Zhou et al., 2019). In the subsequent studies, they converted the difluoro methylene group of **SD-36** to a ketone group to obtain degrader **SD-91** with improved potency both *in vitro* and *in vivo* (Zhou et al., 2021). Kymera Therapeutics chose the similar inhibitor, **SI-109** (Figure 10), but changed the linker attachment site and synthesized a series of degraders based on VHL ligand or CRBN ligand derivatives (Yang et al., 2022). From their disclosed data, the degradation activity of VHL-based degraders was generally better than that of CRBN-based derivatives. However, their recent patent selected CRBN-based ligands for further optimization may due to the large molecular of VHL ligand. It is hypothesized that reducing the molecular weight and the hydrogen bond receptors of E3 ligands may be the trend for PROTACs to be more druggable.

## 3 Conclusions and perspectives

Since PROTAC technology was first identified as a clinical therapeutic strategy, both academia and industry have shown great interest in PROTACs technology. However, PROTACs degraders have relatively more complex chemical structures and biological mechanisms than traditional small molecule drugs, which require efforts in the fields of organic synthetic chemistry and medicinal chemistry.

Although PROTACs technology has shown many advantages over traditional small molecule drugs for antitumor therapy, unlike traditional small molecule drugs, they are mostly regarded as “beyond rule-of-five”. PROTACs molecules have more hydrogen bond donors and acceptors and larger molecular weights, mostly around 1,000 Da. Therefore, the poor membrane permeability and low bioavailability of PROTACs molecules limit its clinical application. In the process of optimizing E3 ligase ligands by medicinal chemists, it is important to ensure a high affinity between the E3 ligase ligands and E3 ubiquitin ligases. The excessive molecular weight of E3 ligands leads to poor membrane permeability. Based on the suitable molecular weight size of CRBN ligand, **ARV-110** and **CFT8634** were rationally designed. In addition, they both introduced F in the aromatic ring of CRBN ligands, probably to improve their druggability. Most of the other CRBN ligands modifications are designed to improve the affinity with E3 ligases or to improve the druggability properties of these degraders in oral PROTACs.

Typically, the POIs based on agonists or antagonists of target proteins, as well as the traditional pharmacological optimization of these small molecules, have been studied in the design and optimization of PROTACs. The optimization of linkers tends to have more rigid structures. However, although more than 600 E3 ligases have been identified, the number of small molecule ligands available to design PROTAC molecules for E3 ligands is rather limited, and only CRBN-based PROTACs have been clinically achieved for oral application (Table 2).

**TABLE 2:** Summary of the chemical properties of E3 ligase ligands in clinical trials.

E3 ligase ligand	E3 ligase	MW	ROA	tPSA	cLog P
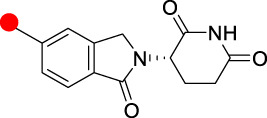	CRBN	244.08	Oral	66.48	0.305
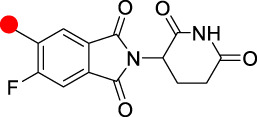	CRBN	276.05	Oral	83.55	0.747
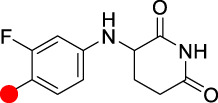	CRBN	222.08	Oral	58.2	1.184
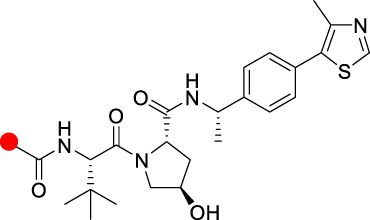	VHL	486.23	I.v	111.1	2.343

Red spot: Linking site.

It is believed that advances in artificial intelligence techniques (protein structure prediction), virtual drug screening, and other technologies will facilitate the discovery of E3 ligands and provide more tools for PROTAC design. These advances will greatly facilitate the transition of PROTACs degraders from being considered as tool molecules to small molecule clinical drug candidates.

## References

[B1] Arnal J. F. Lenfant F. Metivier R. Flouriot G. Henrion D. Adlanmerini M. (2017). Membrane and nuclear estrogen receptor alpha actions: From tissue specificity to medical implications. Physiol. Rev. 97, 1045–1087. 10.1152/physrev.00024.2016 28539435

[B2] Békés M. Langley D. R. Crews C. M. (2022). PROTAC targeted protein degraders: The past is prologue. Nat. Rev. Drug Discov. 21, 181–200. 10.1038/s41573-021-00371-6 35042991 PMC8765495

[B3] Brzezinska A. A. Johnson J. L. Munafo D. B. Ellis B. A. Catz S. D. (2009). Signalling mechanisms for toll-like receptor-activated neutrophil exocytosis: Key roles for interleukin-1-receptor-associated kinase-4 and phosphatidylinositol 3-kinase but not toll/IL-1 receptor (TIR) domain-containing adaptor inducing IFN-beta (TRIF). Immunology 127, 386–397. 10.1111/j.1365-2567.2008.02980.x 19019092 PMC2712107

[B4] Buhimschi A. D. Armstrong H. A. Toure M. Jaime-Figueroa S. Chen T. L. Lehman A. M. (2018). Targeting the C481S ibrutinib-resistance mutation in bruton's tyrosine kinase using PROTAC-mediated degradation. Biochemistry 57, 3564–3575. 10.1021/acs.biochem.8b00391 29851337

[B5] Chen L. Chen Y. Zhang C. Jiao B. Liang S. Tan Q. (2020). Discovery of first-in-class potent and selective tropomyosin receptor kinase degraders. J. Med. Chem. 63, 14562–14575. 10.1021/acs.jmedchem.0c01342 33058680

[B6] Criscitiello C. Guerini-Rocco E. Viale G. Fumagalli C. Sajjadi E. Venetis K. (2022). Immunotherapy in breast cancer patients: A focus on the use of the currently available biomarkers in oncology. Anticancer Agents Med. Chem. 22, 787–800. 10.2174/1871520621666210706144112 34229592

[B7] Czabotar P. E. Lessene G. Strasser A. Adams J. M. (2014). Control of apoptosis by the BCL-2 protein family: Implications for physiology and therapy. Nat. Rev. Mol. Cell Biol. 15, 49–63. 10.1038/nrm3722 24355989

[B8] Davis R. E. Ngo V. N. Lenz G. Tolar P. Young R. M. Romesser P. B. (2010). Chronic active B-cell-receptor signalling in diffuse large B-cell lymphoma. Nature 463, 88–92. 10.1038/nature08638 20054396 PMC2845535

[B9] De Bono J. Mateo J. Fizazi K. Saad F. Shore N. Sandhu S. (2020). Olaparib for metastatic castration-resistant prostate cancer. N. Engl. J. Med. 382, 2091–2102. 10.1056/NEJMoa1911440 32343890

[B10] Drilon A. (2019). TRK inhibitors in TRK fusion-positive cancers. Ann. Oncol. 30, viii23–viii30. viii23-viii30. 10.1093/annonc/mdz282 PMC6859818 31738426

[B11] Duplessis M. Jaeschke G. Kuhn B. Lazarski K. Liang Y. N. Alice Yvonne (2019). Preparation of substituted isoindoline compounds which cause degradation of EGFR and are useful as anticancer agents. U.S. patent NO WO2019149922. Watertown: United States Patent Application Publication.

[B12] Fan J. Qian Y. He W. Liu K. (2021). Bicyclic imide derivative, preparation method thereof, and application thereof in medicine. CN patent NO WO 2021/143822 Al. Shanghai: World Intellectual Property Organization.

[B13] Gao X. Iii H. a. B. Vuky J. Dreicer R. Sartor A. O. Sternberg C. N. (2022). Phase 1/2 study of ARV-110, an androgen receptor (AR) PROTAC degrader, in metastatic castration-resistant prostate cancer (mCRPC). J. Clin. Oncol. 40, 17. 10.1200/JCO.2022.40.6_suppl.017

[B14] Gatchalian J. Malik S. Ho J. Lee D. S. Kelso T. W. R. Shokhirev M. N. (2018). A non-canonical BRD9-containing BAF chromatin remodeling complex regulates naive pluripotency in mouse embryonic stem cells. Nat. Commun. 9, 5139. 10.1038/s41467-018-07528-9 30510198 PMC6277444

[B15] Gollob J. Davis J. Mcdonald A. Rong H. (2022). Irak4 degraders and uses thereof. U.S. patent NO WO 2022/174268 Al. Watertown: World Intellectual Property Organization.

[B16] Gonzalez T. L. Hancock M. Sun S. Gersch C. L. Larios J. M. David W. (2020). Targeted degradation of activating estrogen receptor alpha ligand-binding domain mutations in human breast cancer. Breast Cancer Res. Treat. 180, 611–622. 10.1007/s10549-020-05564-y 32067153

[B17] Han T. Goralski M. Gaskill N. Capota E. Kim J. Ting T. C. (2017). Anticancer sulfonamides target splicing by inducing RBM39 degradation via recruitment to DCAF15. Science 356, eaal3755. 10.1126/science.aal3755 28302793

[B18] Han X. Wang C. Qin C. Xiang W. Fernandez-Salas E. Yang C.-Y. (2019a). Discovery of ARD-69 as a highly potent proteolysis targeting chimera (PROTAC) degrader of androgen receptor (AR) for the treatment of prostate cancer. J. Med. Chem. 62, 941–964. 10.1021/acs.jmedchem.8b01631 30629437

[B19] Han X. Zhao L. Xiang W. Qin C. Miao B. Mceachern D. (2021). Strategies toward discovery of potent and orally bioavailable proteolysis targeting chimera degraders of androgen receptor for the treatment of prostate cancer. J. Med. Chem. 64, 12831–12854. 10.1021/acs.jmedchem.1c00882 34431670 PMC8880306

[B20] Han X. Zhao L. Xiang W. Qin C. Miao B. Xu T. (2019b). Discovery of highly potent and efficient PROTAC degraders of androgen receptor (AR) by employing weak binding affinity VHL E3 ligase ligands. J. Med. Chem. 62, 11218–11231. 10.1021/acs.jmedchem.9b01393 31804827

[B21] He Y. Zhang X. Chang J. Kim H. N. Zhang P. Wang Y. (2020). Using proteolysis-targeting chimera technology to reduce navitoclax platelet toxicity and improve its senolytic activity. Nat. Commun. 11, 1996. 10.1038/s41467-020-15838-0 32332723 PMC7181703

[B22] Henning N. J. Manford A. G. Spradlin J. N. Brittain S. M. Zhang E. Mckenna J. M. (2022). Discovery of a covalent FEM1B recruiter for targeted protein degradation applications. J. Am. Chem. Soc. 144, 701–708. 10.1021/jacs.1c03980 34994556 PMC8928484

[B23] Hershko A. Ciechanover A. (1992). The ubiquitin system for protein degradation. Annu. Rev. Biochem. 61, 761–807. 10.1146/annurev.bi.61.070192.003553 1323239

[B24] Hershko A. Heller H. Elias S. Ciechanover A. (1983). Components of ubiquitin-protein ligase system. Resolution, affinity purification, and role in protein breakdown. J. Biol. Chem. 258, 8206–8214. 10.1016/s0021-9258(20)82050-x 6305978

[B25] Hirsch F. R. Scagliotti G. V. Mulshine J. L. Kwon R. Curran W. J. Wu Y.-L. (2017). Lung cancer: Current therapies and new targeted treatments. Lancet 389, 299–311. 10.1016/s0140-6736(16)30958-8 27574741

[B26] Hohmann A. F. Martin L. J. Minder J. L. Roe J. S. Shi J. Steurer S. (2016). Sensitivity and engineered resistance of myeloid leukemia cells to BRD9 inhibition. Nat. Chem. Biol. 12, 672–679. 10.1038/nchembio.2115 27376689 PMC4990482

[B27] Honda R. Tanaka H. Yasuda H. (1997). Oncoprotein MDM2 is a ubiquitin ligase E3 for tumor suppressor p53. FEBS Lett. 420, 25–27. 10.1016/s0014-5793(97)01480-4 9450543

[B28] Hu J. Hu B. Wang M. Xu F. Miao B. Yang C. Y. (2019). Discovery of ERD-308 as a highly potent proteolysis targeting chimera (PROTAC) degrader of estrogen receptor (ER). J. Med. Chem. 62, 1420–1442. 10.1021/acs.jmedchem.8b01572 30990042

[B29] Huang B. Omoto Y. Iwase H. Yamashita H. Toyama T. Coombes R. C. (2014). Differential expression of estrogen receptor α, β1, and β2 in lobular and ductal breast cancer. Proc. Natl. Acad. Sci. U. S. A. 111, 1933–1938. 10.1073/pnas.1323719111 24449868 PMC3918808

[B30] Igney F. H. Krammer P. H. (2002). Death and anti-death: Tumour resistance to apoptosis. Nat. Rev. Cancer 2, 277–288. 10.1038/nrc776 12001989

[B31] Itoh Y. Ishikawa M. Kitaguchi R. Sato S. Naito M. Hashimoto Y. (2011). Development of target protein-selective degradation inducer for protein knockdown. Bioorg Med. Chem. 19, 3229–3241. 10.1016/j.bmc.2011.03.057 21515062

[B32] Ivan M. Kondo K. Yang H. Kim W. Valiando J. Ohh M. (2001). HIFα targeted for VHL-mediated destruction by proline hydroxylation: Implications for O _2_ sensing. Science 292, 464–468. 10.1126/science.1059817 11292862

[B33] Jaakkola P. Mole D. R. Tian Y. M. Wilson M. I. Gielbert J. Gaskell S. J. (2001). Targeting of HIF-alpha to the von Hippel-Lindau ubiquitylation complex by O2-regulated prolyl hydroxylation. Science 292, 468–472. 10.1126/science.1059796 11292861

[B34] Jackson K. L. Agafonov R. V. Carlson M. W. Chaturvedi P. Cocozziello D. Cole K. (2022). Abstract ND09: The discovery and characterization of CFT8634: A potent and selective degrader of BRD9 for the treatment of SMARCB1-perturbed cancers. Cancer Res. 82, ND09. 10.1158/1538-7445.Am2022-nd09

[B35] Kadoch C. Hargreaves D. C. Hodges C. Elias L. Ho L. Ranish J. (2013). Proteomic and bioinformatic analysis of mammalian SWI/SNF complexes identifies extensive roles in human malignancy. Nat. Genet. 45, 592–601. 10.1038/ng.2628 23644491 PMC3667980

[B36] Kargbo R. B. (2020). PROTAC compounds targeting TRK for use in cancer therapeutics. ACS Med. Chem. Lett. 11, 1090–1091. 10.1021/acsmedchemlett.0c00235 32550985 PMC7294544

[B37] Kargbo R. B. (2019). PROTAC degradation of IRAK4 for the treatment of neurodegenerative and cardiovascular diseases. ACS Med. Chem. Lett. 10, 1251–1252. 10.1021/acsmedchemlett.9b00385 31531192 PMC6746078

[B38] Kawahata I. Fukunaga K. (2020). Degradation of tyrosine hydroxylase by the ubiquitin-proteasome system in the pathogenesis of Parkinson's disease and dopa-responsive dystonia. Int. J. Mol. Sci. 21, 3779. 10.3390/ijms21113779 32471089 PMC7312529

[B39] Khan S. Zhang X. Lv D. Zhang Q. He Y. Zhang P. (2019). A selective BCL-XL PROTAC degrader achieves safe and potent antitumor activity. Nat. Med. 25, 1938–1947. 10.1038/s41591-019-0668-z 31792461 PMC6898785

[B40] Kim K. H. Roberts C. W. (2014). Mechanisms by which SMARCB1 loss drives rhabdoid tumor growth. Cancer Genet. 207, 365–372. 10.1016/j.cancergen.2014.04.004 24853101 PMC4195815

[B41] Klein R. Parada L. F. Coulier F. Barbacid M. (1989). trkB, a novel tyrosine protein kinase receptor expressed during mouse neural development. EMBO J. 8, 3701–3709. 10.1002/j.1460-2075.1989.tb08545.x 2555172 PMC402053

[B42] Kohno T. Matsui T. Enatsu S. (2021). Differences between EGFR exon 19 deletion and exon 21 L858R point mutation, frequently detected EGFR mutations in patients with non-small cell lung cancer, from a molecular biology viewpoint. Gan Kagaku Ryoho 48, 1463–1467. 10.1038/srep31636 34911913

[B43] Komander D. Rape M. (2012). The ubiquitin code. Annu. Rev. Biochem. 81, 203–229. 10.1146/annurev-biochem-060310-170328 22524316

[B44] Kregel S. Wang C. Han X. Xiao L. Fernandez-Salas E. Bawa P. (2020). Androgen receptor degraders overcome common resistance mechanisms developed during prostate cancer treatment. Neoplasia 22, 111–119. 10.1016/j.neo.2019.12.003 31931431 PMC6957805

[B45] Kumar R. Sabapathy K. (2019). RNF4-A paradigm for SUMOylation-mediated ubiquitination. Proteomics 19, e1900185. 10.1002/pmic.201900185 31566917

[B46] Kymera (2022). https://investors.kymeratx.com/news-releases/news-release-details/kymera-announces-positive-results-phase-1-clinical-trial (Accessed Dec 14, 2022).

[B47] Kymera (2022). https://investors.kymeratx.com/news-releases/news-release-details/kymera-therapeutics-doses-first-patients-phase-1-oncology-trials (Accessed Jun 15, 2022).

[B48] Lim K. H. Staudt L. M. (2013). Toll-like receptor signaling. Cold Spring Harb. Perspect. Biol. 5, a011247. 10.1101/cshperspect.a011247 23284045 PMC3579400

[B49] Liu M. Yan M. Lv H. Wang B. Lv X. Zhang H. (2020). Macrophage K63-linked ubiquitination of YAP promotes its nuclear localization and exacerbates atherosclerosis. Cell Rep. 32, 107990. 10.1016/j.celrep.2020.107990 32755583

[B50] Luo M. Spradlin J. N. Boike L. Tong B. Brittain S. M. Mckenna J. M. (2021). Chemoproteomics-enabled discovery of covalent RNF114-based degraders that mimic natural product function. Cell Chem. Biol. 28, 559–566.e15. e515. 10.1016/j.chembiol.2021.01.005 33513350 PMC8052289

[B51] Lyle C. Richards S. Yasuda K. Napoleon M. A. Walker J. Arinze N. (2019). c-Cbl targets PD-1 in immune cells for proteasomal degradation and modulates colorectal tumor growth. Sci. Rep. 9, 20257. 10.1038/s41598-019-56208-1 31882749 PMC6934810

[B52] Mainolfi N. Ji N. Kluge A. F. Weiss M. M. Zhang Y. Zheng X. (2020). Preparation of bifunctional compounds as IRAK degraders and uses thereof. U.S. patent NO WO 2020/113233 Al. Cambridge: World Intellectual Property Organization.

[B53] Martin-Zanca D. Hughes S. H. Barbacid M. (1986). A human oncogene formed by the fusion of truncated tropomyosin and protein tyrosine kinase sequences. Nature 319, 743–748. 10.1038/319743a0 2869410

[B54] Matsui T. Tanizawa Y. Enatsu S. (2021). [Exon 19 Deletion and Exon 21 L858R Point Mutation in EGFR Mutation—Positive Non—Small Cell Lung Cancer]. Gan To Kagaku Ryoho 48 (5), 673–676. 34006711

[B55] Michel B. C. D'avino A. R. Cassel S. H. Mashtalir N. Mckenzie Z. M. Mcbride M. J. (2018). A non-canonical SWI/SNF complex is a synthetic lethal target in cancers driven by BAF complex perturbation. Nat. Cell Biol. 20, 1410–1420. 10.1038/s41556-018-0221-1 30397315 PMC6698386

[B56] Mullard A. (2019). First targeted protein degrader hits the clinic. Nat. Rev. Drug Discov. 21 10.1038/d41573-019-00043-6 30936511

[B57] Nasveschuk C. G. Duplessis M. Ahn J. Y. Hird A. W. Michael R. E. Lazarski K. (2021). Preparation of isoindolinone and indazole compounds for the degradation of EGFR. U.S. patent NO WO2021127561 A1. Watertown: World Intellectual Property Organization.

[B58] Neklesa T. Snyder L. B. Willard R. R. Vitale N. Pizzano J. Gordon D. A. (2019). ARV-110: An oral androgen receptor PROTAC degrader for prostate cancer. J. Clin. Oncol. 37, 259. 10.1200/JCO.2019.37.7_suppl.259

[B59] Neklesa T. Snyder L. B. Willard R. R. Vitale N. Raina K. Pizzano J. (2018). Abstract 5236: ARV-110: An androgen receptor PROTAC degrader for prostate cancer. Cancer Res. 78, 5236. 10.1158/1538-7445.Am2018-5236

[B60] Nunes J. Mcgonagle G. A. Eden J. Kiritharan G. Touzet M. Lewell X. (2019). Targeting IRAK4 for degradation with PROTACs. ACS Med. Chem. Lett. 10, 1081–1085. 10.1021/acsmedchemlett.9b00219 31312412 PMC6627720

[B61] Ohoka N. Morita Y. Nagai K. Shimokawa K. Ujikawa O. Fujimori I. (2018). Derivatization of inhibitor of apoptosis protein (IAP) ligands yields improved inducers of estrogen receptor α degradation. J. Biol. Chem. 293, 6776–6790. 10.1074/jbc.RA117.001091 29545311 PMC5936811

[B62] Ohoka N. Okuhira K. Ito M. Nagai K. Shibata N. Hattori T. (2017). *In vivo* knockdown of pathogenic proteins via specific and nongenetic inhibitor of apoptosis protein (IAP)-dependent protein erasers (SNIPERs). J. Biol. Chem. 292, 4556–4570. 10.1074/jbc.M116.768853 28154167 PMC5377772

[B63] Ohoka N. Tsuji G. Shoda T. Fujisato T. Kurihara M. Demizu Y. (2019). Development of small molecule chimeras that recruit AhR E3 ligase to target proteins. ACS Chem. Biol. 14, 2822–2832. 10.1021/acschembio.9b00704 31580635

[B64] Ohtake F. Fujii-Kuriyama Y. Kato S. (2007). Transcription factor AhR is a ligand-dependcnt E3 ubiquitin ligase. Tanpakushitsu kakusan Koso. Protein, nucleic Acid. enzyme 52, 1973–1979. 10.1038/nature05683 18064888

[B65] Paiva S. L. Crews C. M. (2019). Targeted protein degradation: Elements of PROTAC design. Curr. Opin. Chem. Biol. 50, 111–119. 10.1016/j.cbpa.2019.02.022 31004963 PMC6930012

[B66] Pan Z. Scheerens H. Li S. J. Schultz B. E. Sprengeler P. A. Burrill L. C. (2007). Discovery of selective irreversible inhibitors for Bruton's tyrosine kinase. ChemMedChem 2, 58–61. 10.1002/cmdc.200600221 17154430

[B67] Pei J. Xiao Y. Liu X. Hu W. Sobh A. Yuan Y. (2022). Identification of Piperlongumine (PL) as a new E3 ligase ligand to induce targeted protein degradation. bioRxiv.12 10.1101/2022.01.21.474712

[B68] Pohl C. Dikic I. (2019). Cellular quality control by the ubiquitin-proteasome system and autophagy. Science 366, 818–822. 10.1126/science.aax3769 31727826

[B69] Pulciani S. Santos E. Lauver A. V. Long L. K. Aaronson S. A. Barbacid M. (1982). Oncogenes in solid human tumours. Nature 300, 539–542. 10.1038/300539a0 7144906

[B70] Rebello R. J. Oing C. Knudsen K. E. Loeb S. Johnson D. C. Reiter R. E. (2021). Prostate cancer. Nat. Rev. Dis. Prim. 7, 9. 10.1038/s41572-020-00243-0 33542230

[B71] Remillard D. Buckley D. L. Paulk J. Brien G. L. Sonnett M. Seo H.-S. (2017). Degradation of the BAF complex factor BRD9 by heterobifunctional ligands. Angew. Chem. Int. Ed. 56, 5738–5743. 10.1002/anie.201611281 PMC5967236 28418626

[B72] Robbins D. W. Sands A. T. Mcintosh J. Mihalic J. Wu J. Kato D. (2020). Bifunctional compounds for degrading BTK via ubiquitin proteolytic pathway and their preparation. U.S. patent NO WO2020081450 A1. San Francisco: World Intellectual Property Organization.

[B73] Rodriguez-Gonzalez A. Cyrus K. Salcius M. Kim K. Crews C. M. Deshaies R. J. (2008). Targeting steroid hormone receptors for ubiquitination and degradation in breast and prostate cancer. Oncogene 27, 7201–7211. 10.1038/onc.2008.320 18794799 PMC5573236

[B74] Saadatzadeh M. R. Elmi A. N. Pandya P. H. Bijangi-Vishehsaraei K. Ding J. Stamatkin C. W. (2017). The role of MDM2 in promoting genome stability versus instability. Int. J. Mol. Sci. 18, 2216. 10.3390/ijms18102216 29065514 PMC5666895

[B75] Sabnis R. W. (2021). BRD9 bifunctional degraders for treating cancer. ACS Med. Chem. Lett. 12, 1879–1880. 10.1021/acsmedchemlett.1c00580 34917243 PMC8667060

[B76] Sakamoto K. M. Kim K. B. Kumagai A. Mercurio F. Crews C. M. Deshaies R. J. (2001). Protacs: Chimeric molecules that target proteins to the skp1-cullin-F box complex for ubiquitination and degradation. Proc. Natl. Acad. Sci. U. S. A. 98, 8554–8559. 10.1073/pnas.141230798 11438690 PMC37474

[B77] Sands A. T. Kelly A. (2020). Bifunctional compounds for degrading btk via ubiquitin proteosome pathway. U.S. patent NO WO2021113557. San Francisco: World Intellectual Property Organization.

[B78] Sato S. Aoyama H. Miyachi H. Naito M. Hashimoto Y. (2008). Demonstration of direct binding of cIAP1 degradation-promoting bestatin analogs to BIR3 domain: Synthesis and application of fluorescent bestatin ester analogs. Bioorg Med. Chem. Lett. 18, 3354–3358. 10.1016/j.bmcl.2008.04.031 18448338

[B79] Schapira M. Calabrese M. F. Bullock A. N. Crews C. M. (2019). Targeted protein degradation: Expanding the toolbox. Nat. Rev. Drug Discov. 18, 949–963. 10.1038/s41573-019-0047-y 31666732

[B80] Schneekloth A. R. Pucheault M. Tae H. S. Crews C. M. (2008). Targeted intracellular protein degradation induced by a small molecule: En route to chemical proteomics. Bioorg Med. Chem. Lett. 18, 5904–5908. 10.1016/j.bmcl.2008.07.114 18752944 PMC3175619

[B81] Schneekloth J. S. Jr. Fonseca F. N. Koldobskiy M. Mandal A. Deshaies R. Sakamoto K. (2004a). Chemical genetic control of protein levels: Selective *in vivo* targeted degradation. J. Am. Chem. Soc. 126, 3748–3754. 10.1021/ja039025z 15038727

[B82] Schneekloth J. S. Jr. Fonseca F. N. Koldobskiy M. Mandal A. Deshaies R. Sakamoto K. (2004b). Chemical genetic control of protein levels: Selective *in vivo* targeted degradation. J. Am. Chem. Soc. 126, 3748–3754. 10.1021/ja039025z 15038727

[B83] Schulman B. A. Harper J. W. (2009). Ubiquitin-like protein activation by E1 enzymes: The apex for downstream signalling pathways. Nat. Rev. Mol. Cell Biol. 10, 319–331. 10.1038/nrm2673 19352404 PMC2712597

[B84] Sharma S. V. Bell D. W. Settleman J. Haber D. A. (2007). Epidermal growth factor receptor mutations in lung cancer. Nat. Rev. Cancer 7, 169–181. 10.1038/nrc2088 17318210

[B85] Sievers Q. L. Petzold G. Bunker R. D. Renneville A. Słabicki M. Liddicoat B. J. (2018). Defining the human C2H2 zinc finger degrome targeted by thalidomide analogs through CRBN. Science 362, eaat0572. 10.1126/science.aat0572 30385546 PMC6326779

[B86] Snyder L. B. Flanagan J. J. Qian Y. Gough S. M. Andreoli M. Bookbinder M. (2021a). Abstract 44: The discovery of ARV-471, an orally bioavailable estrogen receptor degrading PROTAC for the treatment of patients with breast cancer. Cancer Res. 81, 44. 10.1158/1538-7445.Am2021-44

[B87] Snyder L. B. Neklesa T. K. Chen X. Dong H. Ferraro C. Gordon D. A. (2021b). Abstract 43: Discovery of ARV-110, a first in class androgen receptor degrading PROTAC for the treatment of men with metastatic castration resistant prostate cancer. Cancer Res. 81, 43. 10.1158/1538-7445.Am2021-43

[B88] Sun X. Gao H. Yang Y. He M. Wu Y. Song Y. (2019). PROTACs: Great opportunities for academia and industry. Signal Transduct. Target Ther. 4, 64. 10.1038/s41392-019-0101-6 31885879 PMC6927964

[B89] Sun Y. Ding N. Song Y. Yang Z. Liu W. Zhu J. (2019). Degradation of Bruton's tyrosine kinase mutants by PROTACs for potential treatment of ibrutinib-resistant non-Hodgkin lymphomas. Leukemia 33, 2105–2110. 10.1038/s41375-019-0440-x 30858551

[B90] Sun Y. Zhao X. Ding N. Gao H. Wu Y. Yang Y. (2018). PROTAC-induced BTK degradation as a novel therapy for mutated BTK C481S induced ibrutinib-resistant B-cell malignancies. Cell Res. 28, 779–781. 10.1038/s41422-018-0055-1 29875397 PMC6028582

[B91] Teo M. Y. Rathkopf D. E. Kantoff P. (2019). Treatment of advanced prostate cancer. Annu. Rev. Med. 70, 479–499. 10.1146/annurev-med-051517-011947 30691365 PMC6441973

[B92] Theodoulou N. H. Bamborough P. Bannister A. J. Becher I. Bit R. A. Che K. H. (2016). Discovery of I-BRD9, a selective cell active chemical probe for bromodomain containing protein 9 inhibition. J. Med. Chem. 59, 1425–1439. 10.1021/acs.jmedchem.5b00256 25856009 PMC7354103

[B93] Tong B. Luo M. Xie Y. Spradlin J. N. Tallarico J. A. Mckenna J. M. (2020). Bardoxolone conjugation enables targeted protein degradation of BRD4. Sci. Rep. 10, 15543. 10.1038/s41598-020-72491-9 32968148 PMC7511954

[B94] Vollmer S. Strickson S. Zhang T. Gray N. Lee K. L. Rao V. R. (2017). The mechanism of activation of IRAK1 and IRAK4 by interleukin-1 and Toll-like receptor agonists. Biochem. J. 474, 2027–2038. 10.1042/BCJ20170097 28512203 PMC5460469

[B95] Waks A. G. Winer E. P. (2019). Breast cancer treatment: A review. JAMA 321, 288–300. 10.1001/jama.2018.19323 30667505

[B96] Wang Y. Jiang X. Feng F. Liu W. Sun H. (2020). Degradation of proteins by PROTACs and other strategies. Acta Pharm. Sin. B 10, 207–238. 10.1016/j.apsb.2019.08.001 32082969 PMC7016280

[B97] Wang Y. Shen Y. Wang S. Shen Q. Zhou X. (2018). The role of STAT3 in leading the crosstalk between human cancers and the immune system. Cancer Lett. 415, 117–128. 10.1016/j.canlet.2017.12.003 29222039 PMC5748258

[B98] Ward C. C. Kleinman J. I. Brittain S. M. Lee P. S. Chung C. Y. S. Kim K. (2019). Covalent ligand screening uncovers a RNF4 E3 ligase recruiter for targeted protein degradation applications. ACS Chem. Biol. 14, 2430–2440. 10.1021/acschembio.8b01083 31059647 PMC7422721

[B99] Wei J. Meng F. Park K. S. Yim H. Velez J. Kumar P. (2021). Harnessing the E3 ligase KEAP1 for targeted protein degradation. J. Am. Chem. Soc. 143, 15073–15083. 10.1021/jacs.1c04841 34520194 PMC8480205

[B100] Winter G. E. Buckley D. L. Paulk J. Roberts J. M. Souza A. Dhe-Paganon S. (2015). Phthalimide conjugation as a strategy for *in vivo* target protein degradation. Science 348, 1376–1381. 10.1126/science.aab1433 25999370 PMC4937790

[B101] Woyach J. A. Furman R. R. Liu T. M. Ozer H. G. Zapatka M. Ruppert A. S. (2014). Resistance mechanisms for the Bruton's tyrosine kinase inhibitor ibrutinib. N. Engl. J. Med. 370, 2286–2294. 10.1056/NEJMoa1400029 24869598 PMC4144824

[B102] Xiang W. Zhao L. Han X. Qin C. Miao B. Mceachern D. (2021). Discovery of ARD-2585 as an exceptionally potent and orally active PROTAC degrader of androgen receptor for the treatment of advanced prostate cancer. J. Med. Chem. 64, 13487–13509. 10.1021/acs.jmedchem.1c00900 34473519 PMC8855934

[B103] Yang B. Zheng X. Zhu X. (2022). Preparation of peptidomimetics as STAT degraders and their uses for treating diseases. CN. patent NO WO2022077010 A1. Watertown: World Intellectual Property Organization.

[B104] Yang F. Jia M. He W. Chen G. He F. Tao W. (2021a). Bicyclic imide derivative, preparation method thereof, and application thereof in medicine. CN. patent NO WO 2021/143822 A1. Shanghai: World Intellectual Property Organization.

[B105] Yang Q. Zhao J. Chen D. Wang Y. (2021b). E3 ubiquitin ligases: Styles, structures and functions. Mol. Biomed. 2, 23. 10.1186/s43556-021-00043-2 35006464 PMC8607428

[B106] Ye Y. Rape M. (2009). Building ubiquitin chains: E2 enzymes at work. Nat. Rev. Mol. Cell Biol. 10, 755–764. 10.1038/nrm2780 19851334 PMC3107738

[B107] Yu H. Pardoll D. Jove R. (2009). STATs in cancer inflammation and immunity: A leading role for STAT3. Nat. Rev. Cancer 9, 798–809. 10.1038/nrc2734 19851315 PMC4856025

[B108] Zhang J. Fu L. Shen B. Liu Y. Wang W. Cai X. (2020a). Assessing IRAK4 functions in ABC DLBCL by IRAK4 kinase inhibition and protein degradation. Cell Chem. Biol. 27, 1500–1509 e1513. 10.1016/j.chembiol.2020.08.010 32888499

[B109] Zhang X. Crowley V. M. Wucherpfennig T. G. Dix M. M. Cravatt B. F. (2019a). Electrophilic PROTACs that degrade nuclear proteins by engaging DCAF16. Nat. Chem. Biol. 15, 737–746. 10.1038/s41589-019-0279-5 31209349 PMC6592777

[B110] Zhang X. Luukkonen L. M. Eissler C. L. Crowley V. M. Yamashita Y. Schafroth M. A. (2021). DCAF11 supports targeted protein degradation by electrophilic proteolysis-targeting chimeras. J. Am. Chem. Soc. 143, 5141–5149. 10.1021/jacs.1c00990 33783207 PMC8309050

[B111] Zhang X. Thummuri D. He Y. Liu X. Zhang P. Zhou D. (2019b). Utilizing PROTAC technology to address the on-target platelet toxicity associated with inhibition of BCL-XL. Chem. Commun. (Camb) 55, 14765–14768. 10.1039/c9cc07217a 31754664 PMC7057339

[B112] Zhang X. Thummuri D. Liu X. Hu W. Zhang P. Khan S. (2020b). Discovery of PROTAC BCL-XL degraders as potent anticancer agents with low on-target platelet toxicity. Eur. J. Med. Chem. 192, 112186. 10.1016/j.ejmech.2020.112186 32145645 PMC7433031

[B113] Zhang X. T. Kang L. G. Ding L. Vranic S. Gatalica Z. Wang Z. Y. (2011). A positive feedback loop of ER-α36/EGFR promotes malignant growth of ER-negative breast cancer cells. Oncogene 30, 770–780. 10.1038/onc.2010.458 20935677 PMC3020987

[B114] Zhao L. Han X. Lu J. Mceachern D. Wang S. (2020). A highly potent PROTAC androgen receptor (AR) degrader ARD-61 effectively inhibits AR-positive breast cancer cell growth *in vitro* and tumor growth *in vivo* . Neoplasia 22, 522–532. 10.1016/j.neo.2020.07.002 32928363 PMC7498667

[B115] Zheng N. Shabek N. (2017). Ubiquitin ligases: Structure, function, and regulation. Annu. Rev. Biochem. 86, 129–157. 10.1146/annurev-biochem-060815-014922 28375744

[B116] Zhou H. Bai L. Xu R. Mceachern D. Chinnaswamy K. Li R. (2021). SD-91 as A Potent and selective STAT3 degrader capable of achieving complete and long-lasting tumor regression. ACS Med. Chem. Lett. 12, 996–1004. 10.1021/acsmedchemlett.1c00155 34141084 PMC8201759

[B117] Zhou H. Bai L. Xu R. Zhao Y. Chen J. Mceachern D. (2019). Structure-based discovery of SD-36 as a potent, selective, and efficacious PROTAC degrader of STAT3 protein. J. Med. Chem. 62, 11280–11300. 10.1021/acs.jmedchem.9b01530 31747516 PMC8848307

